# Cytokine kinetics of Zika virus-infected patients from acute to reconvalescent phase

**DOI:** 10.1007/s00430-015-0445-7

**Published:** 2015-12-24

**Authors:** Dennis Tappe, José Vicente Pérez-Girón, Lorenzo Zammarchi, Jürgen Rissland, Davis F. Ferreira, Thomas Jaenisch, Sergio Gómez-Medina, Stephan Günther, Alessandro Bartoloni, César Muñoz-Fontela, Jonas Schmidt-Chanasit

**Affiliations:** WHO Collaborating Centre for Arbovirus and Haemorrhagic Fever Reference and Research, Bernhard Nocht Institute for Tropical Medicine, Bernhard-Nocht-Str. 74, 20359 Hamburg, Germany; German Centre for Infection Research (DZIF), Partner Site Hamburg–Luebeck–Borstel, Hamburg, Germany; Heinrich Pette Institute, Leibniz Institute for Experimental Virology, Hamburg, Germany; Clinica Malattie Infettive, Dipartimento di Medicina Sperimentale e Clinica, Università Degli Studi di Firenze, Florence, Italy; Institute of Virology, Saarland University Medical Center, Homburg/Saar, Germany; Institute of Microbiology, Federal University of Rio de Janeiro, Rio de Janeiro, Brazil; Section Clinical Tropical Medicine, Department for Infectious Diseases, Heidelberg University Hospital, Heidelberg, Germany

**Keywords:** Zika virus, Arbovirus, Flavivirus, Zika fever, Outbreak, Cytokine

## Abstract

Zika virus is an emerging mosquito-borne flavivirus currently causing large epidemics in the Pacific Ocean region and Brazil. Clinically, Zika fever resembles dengue fever, but is less severe. Whereas the clinical syndrome and laboratory diagnostic procedures have been described, little attention was paid to the immunology of the disease and its possible use for clinical follow-up of patients. Here, we investigate the role of cytokines in the pathogenesis of Zika fever in travelers returning from Asia, the Pacific, and Brazil. Polyfunctional T cell activation (Th1, Th2, Th9, and Th17 response) was seen during the acute phase characterized by respective cytokine level increases, followed by a decrease in the reconvalescent phase.

## Introduction

Zika virus (ZIKV), a mosquito-borne flavivirus, has lately come to international attention owing to outbreaks on Yap Island, Federated States of Micronesia, in 2007 [1] and in French Polynesia beginning in 2013 [2], and a current epidemic after its recent introduction to Brazil [3]. Both epidemics have been attributed to the Asian genotype of ZIKV [2, 3], likely introduced by viremic travelers. The virus is maintained in a natural cycle involving non-human primates and various *Aedes* mosquito species [4]. ZIKV causes a self-limiting, dengue fever (DF)-like disease with an incubation time of up to 10 days. Signs and symptoms consist of rather low-grade fever, myalgia and a maculopapular rash, accompanied by arthralgia and headache, and less often edema, sore throat, and vomiting [1–4]. In contrast to DF, acute Zika fever (ZF) is less severe; headache and malaise are less intense and haemorrhagic and shock complications have not been reported. Conjunctivitis is often present, whereas arthralgia is less pronounced [1]. However, unusual rates of Guillain Barré syndrome (GBS) were reported in French Polynesia due to ZIKV infection [5]. Laboratory changes include transient leukopenia and in some cases thrombocytopenia. Serum aspartate aminotransferase (AST) and alanine aminotransferase (ALT) concentrations may or may not be elevated [6]. Travel-associated infections from both endemic ([6, 7] as examples) and epidemic areas [8, 9] have been documented in non-endemic countries, including the recent report of a patient seen in Italy who became infected in Salvador de Bahia state, Brazil, in the current evolving outbreak.

Whereas the clinical syndrome and laboratory diagnostic procedures, including generic and real-time reverse transcription polymerase chain reactions (RT-PCRs) as well as specific non-commercial serology tests and cross-reactions have been described, little attention was paid to the immunology of the disease and its putative use for clinical follow-up of patients. Here, we analyzed serum cytokine levels of travel-associated infections and demonstrate cytokine concentration changes in acute and recovery (reconvalescent) sera. These changes suggested that strong and multifunctional T cell responses are required for recovery from ZIKV infection.

## Patients and methods

Sera from six patients (three males, three females) aged 31–62 years (median 41 years) were used for this study. Patients had acquired ZIKV infection in Southeast Asia, Polynesia, or Brazil. Blood was drawn from infected patients at different times after disease onset (4–62 days). Sera were classified as either acute (taken ≤10 days after symptom onset) or recovery (taken >10 days after disease onset). ZIKV infection was diagnosed serologically, and in one case also molecularly by RT-PCR [8]; see Table 1 for details. From all sera, individual multiplex cytokine serum analyses (Bio-Rad Laboratories, Munich, Germany) were performed and 20 sera from healthy blood donors were run in parallel. Written informed consent was obtained from each patient.

**Table 1:** Characteristics of patients infected with Zika virus included in the study

Patient number	Age	Sex	Serum taken after disease onset (days)	Travel history	Reference
1	53	Male	31	Thailand	Tappe et al. [6]
2	33	Female	35 and 62	Tahiti	Zammarchi et al. [8]
3	31	Male	33	Tahiti	Zammarchi et al. [8]
4	45	Female	5 and 10	Malaysia	Tappe et al. [7]
5	62	Male	4 and 26	Brazil	Zammarchi et al. [9]
6	37	Female	5	Brazil	Unpublished

## Results

**Fig. 1:**
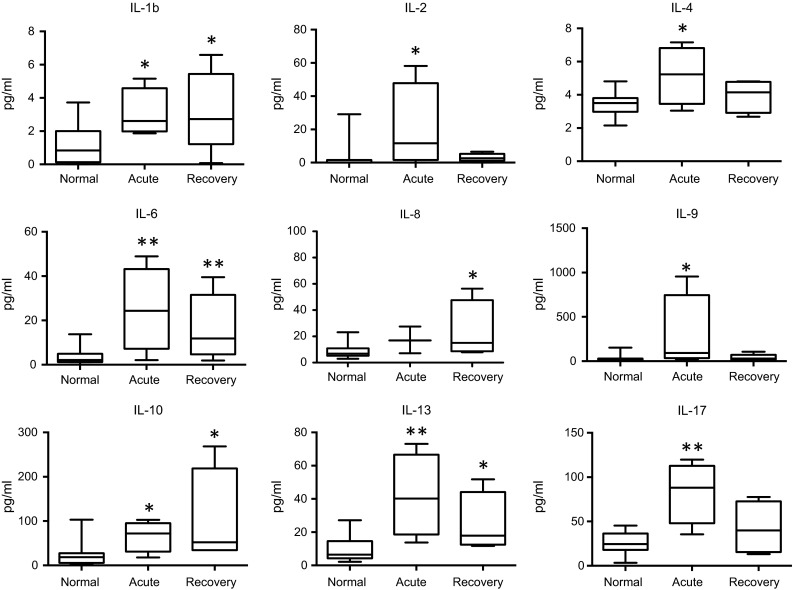
Changes in cytokine levels in the acute and recovery phase of Zika fever. With the exception of IL-8, significant elevations of the serum interleukin concentrations are evident in the early, acute phase of Zika fever. In the recovery phase, IL-1b, IL-8, and IL-10 levels were higher than in the acute phase, whereas levels of the other depicted cytokines were declining. Box-and-whisker plots showing median, upper and lower quartile, minimum, and maximum values. *P < 0.05, **P < 0.01, ***P < 0.001, versus healthy controls (Kruskal–Wallis test)

**Fig. 2:**
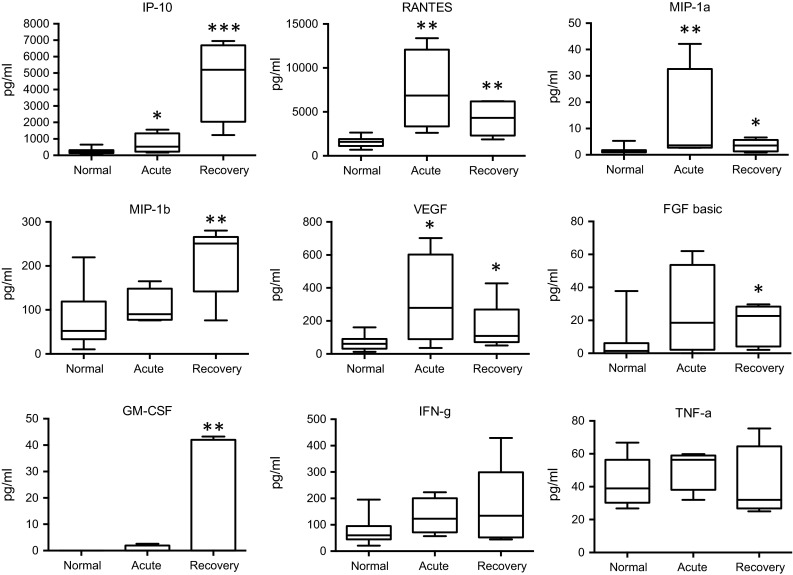
Changes in cytokine and growth factor levels in the acute and recovery phase of Zika fever. RANTES, MIP-1a, and VEGF levels were higher in the acute than in the recovery phase, whereas IP-10, MIP-1b, and GM-CSF reached higher concentrations in the recovery phase than during acute infection. IFN-γ levels showed a non-significant, however, increasing trend over the course of infection, whereas TNF-α concentrations only displayed a non-significant median increase in the acute phase. Box-and-whisker plots showing median, upper and lower quartile, minimum, and maximum values. *P < 0.05, **P < 0.01, ***P < 0.001, versus healthy controls (Kruskal–Wallis test)

In the acute phase of ZF, significant concentration elevations were found for interleukin (IL)-1b, IL-2, IL-4, IL-6, IL-9, IL-10, IL-13, IL-17, as well as for interferon-γ-induced protein 10 (IP-10), regulated on activation, normal T cell expressed and secreted (RANTES), macrophage inflammatory protein 1 alpha (MIP-1a) and vascular endothelial growth factor (VEGF), when compared to normal controls. In the recovery phase, significant increases were demonstrated in the levels of IL-1b, IL-6, IL-8, IL-10, IL-13, as well as of IP-10, RANTES, MIP-1a, MIP-1b, and VEGF, fibroblast growth factor (FGF), granulocyte-macrophage colony stimulating factor (GM-CSF), in comparison with healthy controls. Interferon-γ (IFN-γ) levels showed an increasing trend in the acute and recovery phase (non-significant), whereas tumor necrosis factor-α (TNF-α) concentrations only showed a median increase during the acute phase (also non-significant). Many of the cytokines and factors that were elevated in the acute phase showed a tendency to return to normal levels in the later recovery phase (Figs. 1, 2).

No significant changes, neither in the acute nor in the recovery phase, could be observed for IL-1ra, IL-5, IL-7, IL-12, monocyte chemotactic protein 1 (MCP-1), eotaxin, and platelet-derived growth factor-bb (PDGF-bb; data not shown). There were no significant changes between any of the cytokine levels measured in the acute versus the recovery phase.

## Discussion

In contrast to the clinically similar DF, acute ZIKV infections are less severe and secondary infections are not described owing to the fact that only one serotype of ZIKV exists. After an acute ZIKV infection immunity to re-infection is established for an unknown time span, based on epidemiological evidence from past outbreaks [1, 2]. In the patients studied here, elevation of chemokines was more pronounced than elevation of cytokines. In the recovery phase, cytokine levels generally decreased. A polyfunctional immune activation was seen during the acute phase of ZF, as reflected by elevated cytokine profiles associated with Th1 (IL-2, and non-significantly IFN-γ), Th2 (IL-4, IL-13), Th17 (IL-17), and also Th9 (IL-9) responses. Data on IL-9 functions during human viral infection is scarce, but studies have shown an association of elevated IL-9 levels and severe acute respiratory syncytial virus infections [10] and non-response to treatment of chronic hepatitis C [11], suggesting a modulator role of antiviral immunity. In our study, IL-2, IFN-γ and TNF-α levels were surprisingly lower than expected, contrasting with findings in other arboviral infections [12, 13]. Of note, in our study RANTES is already increased in the acute phase, whereas IP-10 is particularly elevated in the recovery phase. Effective T cell recruitment to infection sites and formation of effector T cells was suggested by both increased RANTES and IP-10 concentrations [14, 15]. Since CCR5 and CxCR3 are chemokine ligands receptors for RANTES and IP-10, respectively, immunophenotyping of peripheral blood T cells of ZIKV-infected patients as well as functional T cell assays should help to further explore the role of RANTES and IP-10 during ZF. Similar to descriptions of patients infected with dengue virus (DENV) [16], a related flavivirus, sera of patients with ZF analyzed in our study also showed increased IL-4, IL-6, IL-8, IL-10, and IP-10 levels. IL-10 levels were also highest in the later (recovery) phase of ZIKV infection, as seen in DF [14]. In contrast to DF, however, concentrations of IL-1ß, IL-2, MCP-1, and VEGF were not decreased in ZF patients. In DF, elevated IL-8 and IL-10 levels were shown to be associated with severe infection [14], a clinical state which has so far not been seen in ZF. No “cytokine storm effects”, as known from DENV infections [17] were demonstrated in the ZF patients in our study. Of note, significantly increased IFN-γ and TNF-α levels were absent in ZF in contrast to DF, pointing toward a Th2 bias in ZIKV infections. In DENV infections, thrombocytopenia correlated strongly with RANTES and VEGF levels [16]. Both RANTES, a chemokine stored in a-granules of platelets, secreted upon platelet activation, and VEGF, a growth factor released by platelets, would be expected to decrease upon thrombocytopenia. In contrast, severe thrombocytopenia is rarely observed in ZF patients and RANTES and VEGF did not show decreased levels when compared with the control group. It remains to be elucidated whether ZIKV-induced GBS [5] might be associated with different cytokine responses than changes in non-GBS ZIKV-infected patients.

Due to the small number of ZF patients examined, this study and the strength of the observations are limited. We have analyzed cytokine levels over time in returning travelers to Europe, and it is unknown whether different human ethnic groups might show different cytokine responses as described for DF [18]. Recent ZF epidemics were only caused by the Asian genotype of ZIKV, which is the same genotype also seen in travel-related infections from outbreak areas. Thus, the cytokine data presented here are linked to an infection with the Asian genotype. As a restriction of the study, functional T cell tests (i.e., stimulation with ZIKV antigen followed by intracellular cytokine staining) have not been performed. Future studies will address lymphocyte and cytokine responses in more detail, allowing proper conclusions of Th1- and Th2-biased cell responses. Despite showing rather preliminary data, to our knowledge, this is the first study investigating immune parameters in acute ZF.
